# A Survey of Handwashing Knowledge and Attitudes among the Healthcare Professionals in Lahore, Pakistan

**DOI:** 10.7759/cureus.1089

**Published:** 2017-03-09

**Authors:** Ahsan Zil-E-Ali, Mohsin A Cheema, Muhammad Wajih Ullah, Hamzah Ghulam, Mariam Tariq

**Affiliations:** 1 Surgery, Pakistan Institute of Medical Sciences, Islamabad, Pakistan; 2 Department of Physiology, FMH College of Medicine; 3 Department of Internal Medicine & Critical Care, Fatima Memorial Hospital; 4 Medical Student-4, FMH College of Medicine

**Keywords:** hand hygiene, healthcare worker

## Abstract

**Objective:**

To evaluate the knowledge and attitudes towards handwashing of clinical year medical and dental students and health-care professionals (HCPs) working in the departments of medicine, surgery, dentistry, nursing, and physiotherapy in Lahore, Pakistan.

**Methodology:**

This was a cross-sectional survey conducted during May and June 2016. After approval from the institutional review board, a modified form of the World Health Organization (WHO) Hand Hygiene Knowledge Questionnaire for Healthcare workers that included 18 items was sent to 400 clinical year students and HCPs from six medical colleges and affiliated hospitals of Lahore. The data obtained was entered and analyzed by using IBM SPSS version 20 (IBM, NY, USA). Chi-square was used as the test of significance. A p*-value *of <0.05 was considered statistically significant for all purposes.

**Results:**

The response rate was 79%. Less than half of the respondents (149, 47.9%) were satisfied with their knowledge regarding hand hygiene. Statistically significant associations of various groups of HCPs were observed with their satisfaction regarding knowledge about hand hygiene (p-value = 0.022), their awareness of the proper technique required for handwashing proposed by the WHO (p-value = 0.001), and their awareness about other preventive techniques proposed by the WHO and Centers for Disease Control (CDC) (p-value = 0.021).

**Conclusions:**

The majority of the clinical year students and HCPs were not satisfied with their knowledge regarding hand hygiene. HCPs working in different departments have varying knowledge and attitudes towards hand hygiene. Females were found to be more satisfied with their handwashing practices. Teaching proper technique of handwashing to medical students and starting refresher courses regarding hand hygiene for HCPs are dire needs. The WHO-recommended guidelines should not only be taught but also implemented in the medical field as poor hand hygiene techniques have led to the spread of many diseases around the globe.

## Introduction

A worldwide campaign on hand hygiene by the World Health Organization (WHO) is an important initiative for a basic protection from various infectious diseases. In 2009, the WHO started a campaign, ‘Save Lives: Clean Your Hands,’ which encompassed the handwashing techniques and a simple way of managing drug-resistant bacteria [1].

Every year millions of patients around the world are affected by infections that are transmitted by the health-care professionals (HCPs) [2-3]. Most of these infections can be prevented through a simple precautionary measure of proper hand wash [4]. Unfortunately, compliance with the hand hygiene guidelines provided by the WHO is usually poor among HCPs, and the hand hygiene equipment is not up to standards [5].

In Pakistan, infectious diseases are a major burden for which the WHO initiated the Hand Hygiene campaign in Pakistan Institute of Medical Sciences (PIMS) in 2007. As a result of this campaign, prevention of health-care associated infections (HCAIs) has become a high priority patient agenda of PIMS.

The medical and dental students including interns are always neglectful about their knowledge and perception of the WHO health guidelines of hand hygiene despite the fact that they spend an ample time in clinical settings. However, the compliance of HCPs to hand hygiene is unknown [6]. The WHO presented a set of tools for evaluation and feedback of the hand hygiene campaign efficacy; a knowledge questionnaire was also proposed. This 21 set questionnaire covered the knowledge of health-care workers regarding hand hygiene [7].

Our survey aimed to find how much students and HCPs know and practice WHO handwashing techniques as there is no reliable data collected before. The questionnaire used in this study covers the perceptions as well as various hygiene practices by HCPs in clinical setting and also in common routine. It aims that proper strategies should be made for compliance and proper teaching of handwashing techniques proposed by the WHO [8]. ​

## Materials and methods

The study was conducted on the clinical year students and HCPs in tertiary care settings about hand hygiene and knowledge about the WHO handwashing guidelines. This cross-sectional study was carried out in Lahore on 315 clinical year medical and dental students and doctors from six medical colleges during May and June 2016. The study population was stratified into three major groups, i.e. medical students, doctors, and allied HCPs that also included the nursing staff. The study participants were included by simple probability sampling done in the stratified groups. The students were explained about the nature and content of the study.

A self-administered questionnaire comprising 18 questions was distributed among the students and doctors to assess perception of hand hygiene and knowledge of handwashing guidelines proposed by the WHO. The questionnaire was a modified version of the standardized questionnaire of the WHO [7]. A pilot survey was conducted using this modified version of the questionnaire and internal consistency was tested before its application on our designated sample. All data was entered in SPSS 20 (IBM, NY, USA), and categorical data was expressed on frequency tables, and test of significance was applied. Chi-square was used as the test of significance. The p-value of 0.05 was considered to be statistically significant.

Ethical clearance was obtained from the institutional review board (IRB) of Fatima Memorial Hospital College of Medicine and Dentistry, Lahore, and it was made sure that the approach of researchers should be non-coercive. In addition, a written consent was obtained from each respondent before the questionnaire was filled.

## Results

Four hundred individuals were asked to fill the questionnaire, of which only 315 responded. A response rate of 79% was recorded. More than half of the respondents were females (165, 52.4%). Among respondents, 184 (58.4%) were doctors, 111 (35.2%) were students, and 20 (6.3%) were allied health professionals who included physiotherapists and nurses. Significantly, higher proportion of female than male respondents were satisfied with their knowledge about hygiene, i.e., 90 (54.5%) compared to 59 (39.3%) (p-value = 0.036). The females had proper knowledge of the procedure of handwashing and they followed those guidelines. However, a significantly higher proportion of females (35, 21.2%) than males (20, 13.3%) also responded that either they did not know or have never heard about the proper technique required for handwashing proposed by the WHO (p-value = 0.013). Similarly, a significantly lower proportion of females (22, 13.3%) and males (nine, 6.0%) responded that they know about the other preventive techniques proposed by the WHO and Centers for Disease Control (CDC) as compared to other responses in the questionnaire (Table 1). 

**Table 1:** Distribution of various responses regarding the practices and perceptions of hand hygiene of the healthcare professionals

Questions	Responses				
How often do you wash your hands in a non-hospital setting?	Before eating	Before & after eating	Rarely	After using the toilet	Before and after eating, after using the toilet
	41 (13%)	54 (17.1%)	2 (0.6%)	39 (12.4%)	179 (56.8%)
How do you prefer to touch/examine the patient?	At times with gloves but usually bare handed		Depends on the case	Gloves	Bare handed
	28 (8.9%)		129 (41.0%)	117 (37.1%)	41 (13%)
In an operation theater setting how often do you practice the right technique for washup?	Almost every time		Quite often	Always	Never
	179 (56.8%)		77 (24.4%)	44 (14 %)	15 (4.8%)
Are you satisfied with your knowledge about hygiene?	Satisfactory	To some extent	Yes	No	Need to know more
	149 (47.3%)	106 (33.7%)	42 (13.3%)	8 (2.5%)	10 (3.2%)
Are you aware of the proper technique required for hand washing proposed by the World Health Organization?	Never heard about it		Yes	No	Not much
	20 (6.3%)		201 (63.8%)	35 (11.1%)	59 (18.7%)
Are you aware of the other preventive techniques proposed by the WHO and CDC?	I don't know about this		Not much	Heard about it	Yes a lot
	138 (43.8%)		91 (28.9%)	55 (17.5%)	31 (9.8%)
Were you ever taught about handwashing at your medical school?	Just an overview		Yes	Never	Not much
	43 (13.7%)		162 (51.4%)	90 (28.6%)	20 (6.3%)

The categories of HCPs were found to be significantly associated with responses to several questions asked in the survey. A significant association was found between the health professionals and the timing of handwash (p-value = <0.001) with maximum number of students (47, 42.3%), doctors (119, 64.7%), and allied health professionals (13, 65%) responding that they wash hands before and after eating as well as after using the toilet. The use of gloves during patient examination was also associated with different health professionals (p-value = 0.001). The response to the question regarding the right technique for washup in an operation theatre setting was also significantly associated with HCPs (p-value = <0.001), again with the majority of students (50, 45.0%), doctors (120, 65.2%), and allied health professionals (nine, 45%) responding that they “always” employ the right technique of washup in an operation theatre setting. Significant associations of HCPs with satisfaction regarding knowledge about hygiene (p-value = 0.022), awareness of the proper technique required for handwashing proposed by the WHO (p-value = 0.001), and awareness about other preventive techniques proposed by the WHO and CDC (p-value = 0.021) were also observed.

## Discussion

Our study scoped important hygiene perceptions and comparative analysis among the HCPs. Out of the 400 selected participants, only 315 responded, stating a reluctance or lesser knowledge about this aspect of health. Females were found to be more satisfied with their knowledge regarding hand hygiene, which could be a gender-selective behavior. However, a significantly high number of females had never heard about the proper technique. These two non-parallel results state a confused status of the females or it could be possible that they considered their knowledge about hand hygiene more satisfactory as compared to recommended protocols. It was encouraging to see that the timing of the handwash was adequate and was equal to proposed standards. The use of gloves and its association with health professionals was also a positive finding. In the operation theatre setting, the majority of doctors knew the standardized hand hygiene and washup methods, although the students and the allied professionals knew less about it. Another important finding was that the majority of doctors and allied HCPs were punctual about hand hygiene before and after eating, though the percentage of students regarding this practice was less than half. This could be due to hasty behavior or an ignorant psychological status of the students.

Hand hygiene is an important component of clinical practice, as hands can serve as a primary thoroughfare for infectious agents to be transmitted among the patients through HCPs. The first Global Patient Safety Challenge, a project initiated by the WHO, focused on the improvement of hand sanitation practices that can present strong patient safety culture. There can be a marked reduction of various diseases by making this recommendation into a routine custom among the medical practitioners [9].

The WHO World Alliance for Patient Safety, Switzerland presented religion and culture, as important parameters for increasing people's compliance with hand hygiene measures. Their analysis was based on extensive literature and extended through seven major religions of the world. The possibility of less application of alcohol-based products can be because of the prohibition of alcohol in some religions. Although the study explained that religion and culture of various ethnic regions can promote hand hygiene in a multimodal strategy, this can easily overcome the barriers so far maintained among the HCPs [10]. This could be used as a model throughout the world irrespective of professionals and can directly reduce the infectious diseases to a remarkable number.

Apart from our study showing some satisfying facts and some data not much inclusive, a study in Sao Paulo, Brazil documented that despite a very comprehensive infrastructure, there was less adequate sanitation by the health professionals. Alcohol use was not seen despite its availability. This study was observed thoroughly with very disappointing results [11]. In comparison, our HCPs were statistically more practical in maintaining hygiene and abiding by the proposed protocols.

The CDC encourages handwashing with soap and water, as it is more effective than alcohol-based hand rub (ABHR) gel in removing C. difficile spores, one of the known pathogens in operation theatre setting and hospital. This clears that most of the doctors knew hand hygiene which is promising to see among our study participants [12-13].

A study by Anargh conducted in Pune, India stated that the majority of HCPs preferred soap and water for hand sanitation [14]. Feather, et al. carried a study for the hand hygiene practices of 187 candidates during the final MBBS OSCE (Objective Structured Clinical Examination) at The Royal London Hospital School of Medicine and Dentistry in the UK and found that only 8.5% of candidates washed their hands after patient contact. This practice was not very satisfactory, plus at this undergraduate level, this practice shows reluctance or lack of knowledge, which establishes the poor basis for the young physicians [15].

As compliance for handwashing is low among HCPs in tertiary care hospital, proper availability of resources and multidimensional programs should be carried out to improve hand hygiene practices, as was mentioned in CDC's Healthcare Infection Control Practices Advisory Committee (HICPAC) comprehensive guidelines for hand hygiene (HH) in healthcare settings in 2002 [16].

In this study, response bias of the participants might have affected the result with a possibility of over or underestimation of compliance. Also, it would have been better if the participants were observed during their handwashing in the clinical setting but then addressing Hawthorne effect could have confounded our data, as observing their practices without taking them into confidence could bring various aspects of ethical considerations in motion. Another aspect is that we can keep all the study participants under observation for a time duration and interview them at multiple times in various clinical settings, but the practice of hand hygiene among the HCPs may differ from place to place. Thus, the validity can be increased by comparing it with HCPs in urban practice and the health facilities in rural and the underprivileged localities.

## Conclusions

Hand hygiene is an important practice in health care. An adequate knowledge about this and its practicality among the HCPs can directly affect the health care system. The study had its significance in this regard as we saw major responses from the health professionals and students. It was concluded that there is a dire need to improve hand hygiene conditions and educate them further according to the WHO guidelines. The response from the survey also found that females had a greater perception about hand hygiene procedure. A significant finding was that there was not an adequate knowledge of proper procedure among males. Our study concluded that females were more satisfied with their hand hygiene despite the lesser knowledge about it. Standardized time for washing hands and proper usage of gloves among the study participants were passable. Though the doctors and the HCPs were with the best knowledge of washup technique, the students were lacking in it. Overall, the perceptions and the practices of these HCPs were satisfactory to sustain the health care system.
